# Ontology for the Asexual Development and Anatomy of the Colonial Chordate *Botryllus schlosseri*

**DOI:** 10.1371/journal.pone.0096434

**Published:** 2014-05-01

**Authors:** Lucia Manni, Fabio Gasparini, Kohji Hotta, Katherine J. Ishizuka, Lorenzo Ricci, Stefano Tiozzo, Ayelet Voskoboynik, Delphine Dauga

**Affiliations:** 1 Department of Biology, University of Padova, Padova, Italy; 2 Department of Biosciences and Informatics, Faculty of Science and Technology, Keio University, Kouhoku-ku, Yokohama, Japan; 3 Institute for Stem Cell Biology and Regenerative Medicine, and Hopkins Marine Station, Stanford, California, United States of America; 4 Centre National de la Recherche Scientifique, Sorbonne Universités, Université Pierre et Marie Curie (University of Paris 06), Laboratoire de Biologie du Développement de Villefranche-sur-mer, Observatoire Océanographique, Villefranche-sur-mer, France; 5 Bioself Communication, Marseille, France; Laboratoire Arago, France

## Abstract

Ontologies provide an important resource to integrate information. For developmental biology and comparative anatomy studies, ontologies of a species are used to formalize and annotate data that are related to anatomical structures, their lineage and timing of development. Here, we have constructed the first ontology for anatomy and asexual development (blastogenesis) of a bilaterian, the colonial tunicate *Botryllus schlosseri*. Tunicates, like *Botryllus schlosseri*, are non-vertebrates and the only chordate taxon species that reproduce both sexually and asexually. Their tadpole larval stage possesses structures characteristic of all chordates, *i.e.* a notochord, a dorsal neural tube, and gill slits. Larvae settle and metamorphose into individuals that are either solitary or colonial. The latter reproduce both sexually and asexually and these two reproductive modes lead to essentially the same adult body plan. The *Botryllus schlosseri* Ontology of Development and Anatomy (BODA) will facilitate the comparison between both types of development. BODA uses the rules defined by the Open Biomedical Ontologies Foundry. It is based on studies that investigate the anatomy, blastogenesis and regeneration of this organism. BODA features allow the users to easily search and identify anatomical structures in the colony, to define the developmental stage, and to follow the morphogenetic events of a tissue and/or organ of interest throughout asexual development. We invite the scientific community to use this resource as a reference for the anatomy and developmental ontology of *B. schlosseri* and encourage recommendations for updates and improvements.

## Introduction

Ascidiacea is a class of marine organisms belonging to the chordate subphylum Tunicata, which is a sister group of Vertebrata [1]–[3]. Ascidians species encompass solitary and colonial forms, and can adopt different reproductive strategies to develop an adult body through different ontogenetic pathways. Following embryonic development, a swimming larva with structural chordate characteristics - a notochord, dorsal neural tube, segmented musculature and gill slits - settles on a suitable substrate and metamorphoses into a sessile filter feeding zooid, losing most of its morphological chordate characteristics [4]. After metamorphosis, the oozooid (zooid derived from a fertilized egg) begins a lifelong, recurring asexual reproduction through blastogenesis or budding, which eventually gives rise to genetically identical individuals, the blastozooids (zooids derived from blastogenesis) [5], [6].

Among colonial ascidians, the species *Botryllus schlosseri* has been described and comprehensively studied for more than half a century [5]–[7]. In *B. schlosseri*, the larva already bears one bud, which grows on one side of the newly settled oozooid and forms the first adult blastozooid. The latter is then able to produce several lateral buds. This modality of blastogenesis is called “palleal” budding. The colony of *B. schlosseri* is characterized by synchronized waves of budding cycles accompanied by regression and resorption of the filtering adults. After several blastogenetic cycles, the colony organizes itself in star-shaped systems of 5–15 clonal blastozooids arranged around a common cloacal, excurrent siphon. All blastozooids are embedded in a common tunic and connected by a vascular system. Each adult blastozooid bears one to several buds on which the new generation of young budlets is developing (reviewed in [5]).

In the last couple of decades *B. schlosseri* has emerged as a model because of its extraordinary regenerative plasticity [8]–[14], and its peculiar allorecognition system, which makes it an ideal archetype to explore the evolutionary origin of an adaptive immune system [15]–[22]. The potential to track in vivo long-lived adult germ and somatic stem cells has advanced *B. schlosseri* as a model to study the biology of stem cell parasitism, chimerism [23]–[32], and ageing related phenomena [33]–[37]. Due to its ability to maintain colony homeostasis, *B. schlosseri* has also been studied as a reliable model for *in vivo* studies of apoptosis and phagocyte dynamics [38]–[42].

Because of these intrinsic characteristics and the potential uses of this model, numerous efforts have been combined to develop and adapt techniques and protocols to facilitate the study of *B. schlosseri*. The anatomy and histology of the blastozooid has been described [40], [43]–[50]; and basic molecular tools have been developed, including development of genetically defined lines [51], [52]; morpholino and siRNA knockdown [9], [13], [18], [19], [53], [54], transplantation and *in vivo* tracking of cells [23], [27], [30], [41]. In addition, vast transcriptome datasets have been generated [3], [55]–[57], and genome has been assembled [3]. From these databases, the initial gene ontology, annotation and assignment to chromosomes were presented, exponentially enriching the accessibility to molecular markers.

The community of scientists interested in *B. schlosseri* as a model organism has increased due to the availability of tools and the applicability of topics like stem cell and regenerative biology, ageing and immunity. However, despite the solid morphological background available, the distinctiveness of the asexual development of *B. schlosseri* lacks a straightforward and comprehensive key. In an effort to standardize and define the life cycle of this organism, a common staging system has been adopted by the Tunicate community, published in a comprehensive review by Manni et al. [5]. Based on these premises, and the knowledge collected in the last decades we established the *Botryllus schlosseri* Ontology of Development and Anatomy (BODA). BODA presents the first ontology for an asexual model of development in bilaterians and particularly in chordates. It follows the existing examples of the embryonic anatomy and developmental ontologies of the solitary ascidian species: *Ciona intestinalis, Ciona savygni, Halocynthia roretzi*, and *Phallusia mamillata* ¸ all widely used as models for embryology [58]–[61].

The *Botryllus* ontology is presented as an open and implementable automated retrieval system that can be integrated with available biological information (for example gene expression obtained by *in situ* hybridization) and improved and updated upon the release of new data.

## Materials and Methods

**Figure 1:**
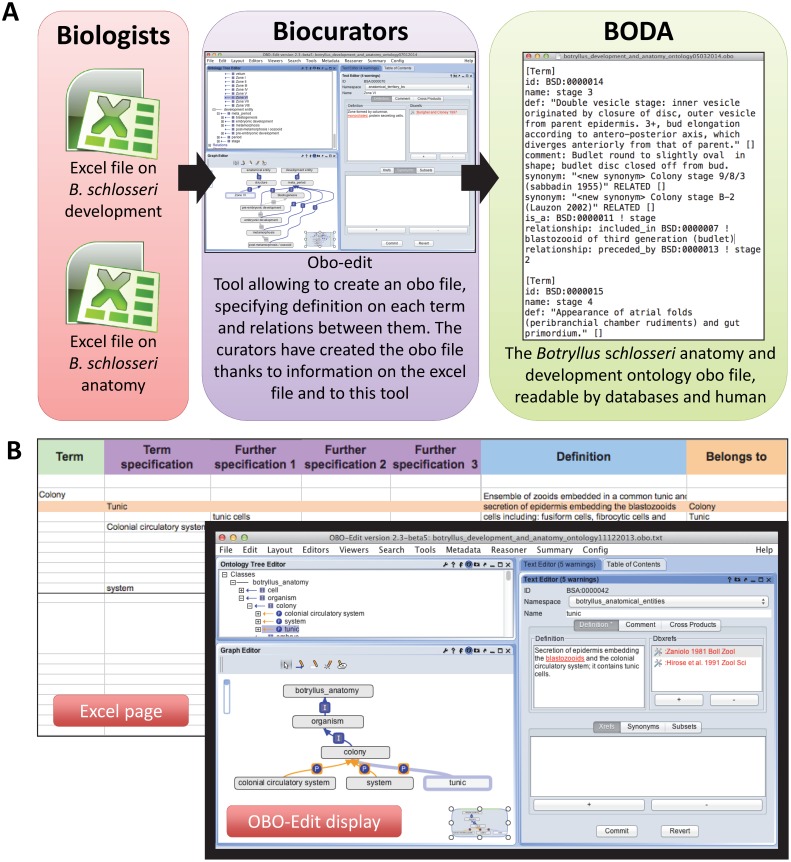
The workflow adopted to build BODA. **A.** Schemes illustrating the workflow adopted to build BODA requiring strict collaboration between experts in *B. schlosseri* biology and biocurators. **B.** An Excel page and its corresponding display on OBO-edit. In the latter, you can see different visualization of the relationship “tunic is part of colony” (left panel of the OBO-Edit display); the definition and references of the term “tunic” are in the right panel (where you can also find synonyms, comments, etc, where introduced).

The BODA types, synonyms, definitions and information about developmental events and anatomical entities have been accumulated from textbooks, journals and scientific observations. This information has been collected and formatted in two excel files: one file on anatomy, the other on development. BODA was built in OBO format by using the open source graphical ontology editor OBO-edit ([62], Fig. 1). Top-level anatomical structures, development events and corresponding definition are based on main nodes of the *Ciona intestinalis* ontology [63].

### Life Cycle of *B. schlosseri*

**Figure 2:**
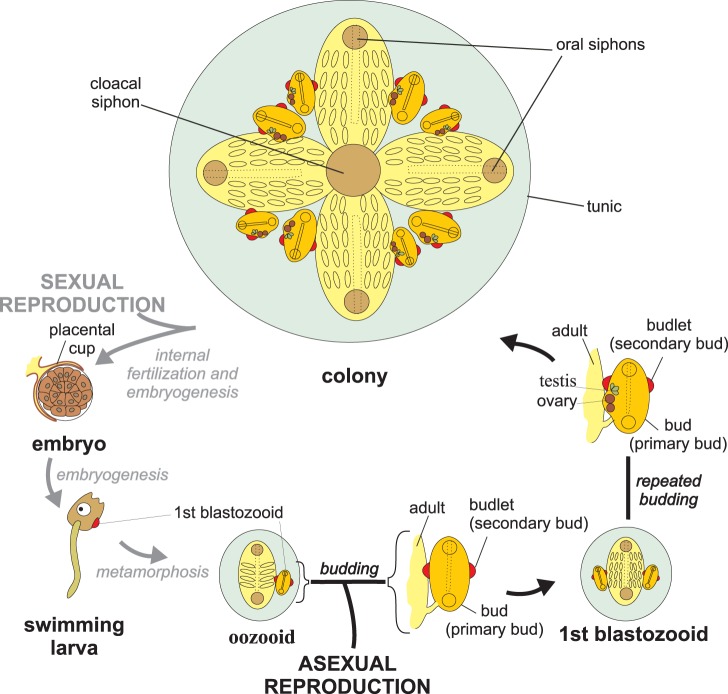
Scheme of *Botryllus schlosseri* life cycle. Ozooid and blastozooids are shown in a dorsal view. The colony is represented as formed by a single system of four adult blastozooids each bearing two buds, that in turn bear two budlets; all the other drawings represent zooids oriented with the anterior region up; modified from [95].

**Figure 3:**
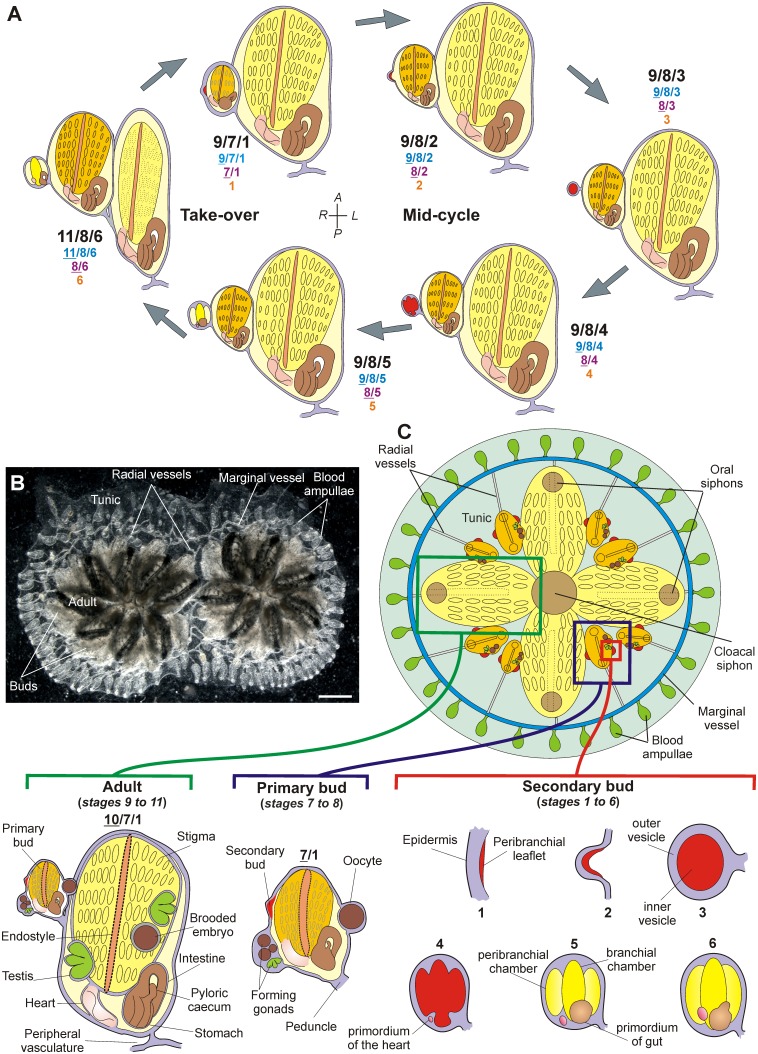
Blastogenetic cycle of *B. schlosseri*. **A.** Scheme showing the colonial cycle and the corresponding stages of blastozooid development (sketched in ventral view, irrespective of the true reciprocal per-lateral orientation between the three generations). Colony stages are indicated by a combination of three numbers separated by slashes (black); stages of blastozooids of first, second and third generation are indicated in blue, purple and orange, respectively. The blastozooid begins its life as a small secondary bud at stage 1, becomes a primary bud passing from stage 6 to 7/1, and adult passing from stage 8/6 to 9/7/1. Its regression occurs at stage 11/8/6. The take-over represents the colonial phase in which changes of generation occurs. In blastozooids, anterior at top and posterior at bottom (modified from [40]). **B.** Ventral view of a colony of *B. schlosseri*, constituted of two systems. Scale bar: 1 mm. **C.** Scheme of a system (dorsal view) and details of its blastozooids (ventral view). In blastozooids, anterior at top, posterior at bottom and irrespective of the true reciprocal per-lateral orientation between the three generations (modified from [5]).

The ascidian *Botryllus schlosseri* (family Styelidae, order Stolidobranchiata) forms colonies composed of several zooids embedded in a common tunic (Figs. 2, 3). In adult zooids mature eggs ovulate and move into the peribranchial chamber, where they are fertilized by the sperm of another colony. Zooids are sequentially hermaphroditic, *i.e.* testes mature later than eggs so self-fertilization is usually prevented. Embryos develop in the peribranchial chamber and are held *in situ* by a placental cup (Fig. 2). After about a week of gestation (20°C; [46]), a swimming tadpole larvae is released through the atrial aperture of the parental zooid. Within 36–48 hours, the anterior papillae adheres to a suitable substrate, resorbs the tail, and metamorphoses into a fully functional oozooid, approximately 0.5 mm in length. Through blastogenesis, the oozooid, which represents the founder individual (Fig. 2), generates a colony composed of zooids, all of which share the same genotype, and are in fact clones.

In a colony, three blastogenetic generations usually coexist: the adult zooids, their buds, also called primary buds, and the budlets, *or* secondary buds, sprouting from the primary buds. The development of buds and budlets is highly synchronized within the colony (Fig. 3): during the stage referred to as take-over, all adults are synchronously resorbed and replaced by primary buds, while secondary buds become primary buds and give rise to a new budlet generation [14]. This cyclical colonial phase represents the generation change. During take-over old zooids contract and undergo massive, diffuse apoptosis of their tissues [38]. The blastogenetic cycle starts with the opening of the siphons of the new adult zooids and ends with the conclusion of the take-over phase, when the next blastogenetic generation reaches functional maturity. This time interval, in which buds and budlets gradually grow, takes approximately one week at 18–19°C [6], [64].

## Results

**Figure 4:**
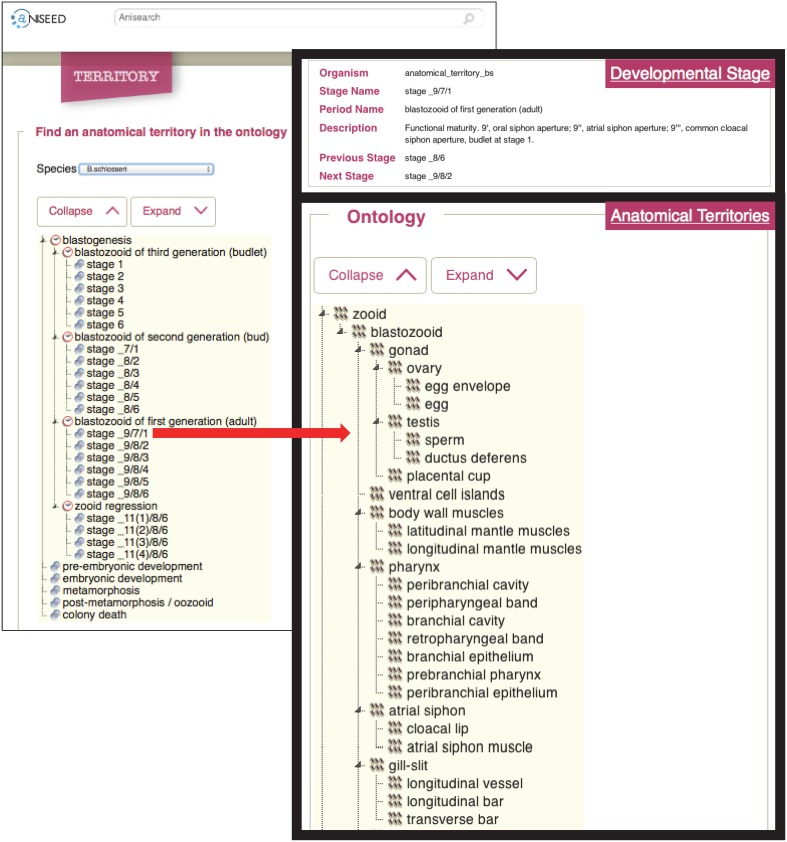
Schemes illustrating BODA in NISEED. BODA can be easily browsed using the NISEED platform (www.aniseed.cnrs.fr), which represents the reference portal in the tunicate community. It is possible to access the ontologies through the menu “anatomy”, select the stage of interest and access to the corresponding definitions and relations.

BODA can be downloaded as OBO file from OBO Foundry portal [65], from the Ontology Bioportal (http://bioportal.bioontology.org/ontologies/BODA) [66] or from the Tunicate Portal (http://www.tunicate-portal.org/wordpress/?page_id=145). Users can also browse it directly using the ontology bioportal and/or NISEED platform (Fig. 4).

The BODA tree is divided in two “classes”: an **anatomical entity** (BSA:0000034) and **a development entity** (BSD:0000034). Identification codes are respectively indicated as “BSA”, if entities are referred to the *B. schlosseri* anatomy, or “BSD”, if entities are referred to the *B. schlosseri* development.

From now on, each entity is written in bold when introduced for the first time, relations between entities in italics, with identification codes between brackets and entity definitions between quotation marks.

Since the oozooid, *i.e.* the **zooid** (BSA:0000040) derived from the metamorphoses of the tadpole larva, is the founder of the **colony** (BSA:0000041), some colonial anatomical entities of the ontology find their origin (*i.e.*, *develops from*) directly from oozooid or larval structures. For instance, the **colonial circulatory system** (BSA:0000044) *is a* structure *part of* the colony. The system originates from the larval **blood ampullae** (BSA:0000045) and extends into the **tunic** (BSA:0000042) forming the **marginal vessel** (BSA:0000047) and **radial vessels** (BSA:0000048) during metamorphosis and oozooid life.

In healthy colonies, and in normal conditions, budding always occurs on stereotyped regions of the **peribranchial epithelium** (BSA:0000115) of the parental zooid: anterior right side, anterior left side, posterior right side and posterior left side. In general, the anterior-right side has a higher blastogenic potential than the other ones [5], [6], [67]–[69]. In order to stage a colony, only the development of the anterior-right bud was considered for the construction of the ontology of the anatomy and development.

### The Development Ontology of *Botryllus schlosseri* Blastogenesis

**Figure 5:**
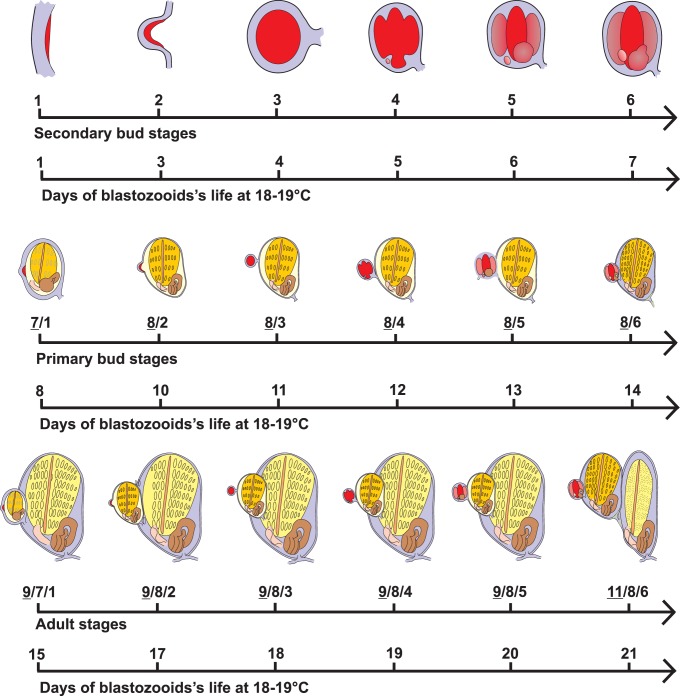
Developmental timetable of *B. schlosseri* blastozooid. The life of each blastozooid lasts about three weeks at 18–19°C: the first week involves the maturation of the budlet, from its appearance to organogenesis mostly concluded; the second week, preceeded by a change of generation during which the budlet becomes a bud and form a new budlet, sees the bud growing, the heart beating, and the organs cytodifferentiation; the third week, again preceded by a change of generation during which the bud become adult, is characterized by filter feeding activity. See the text for details about staging method.

**Figure 6:**
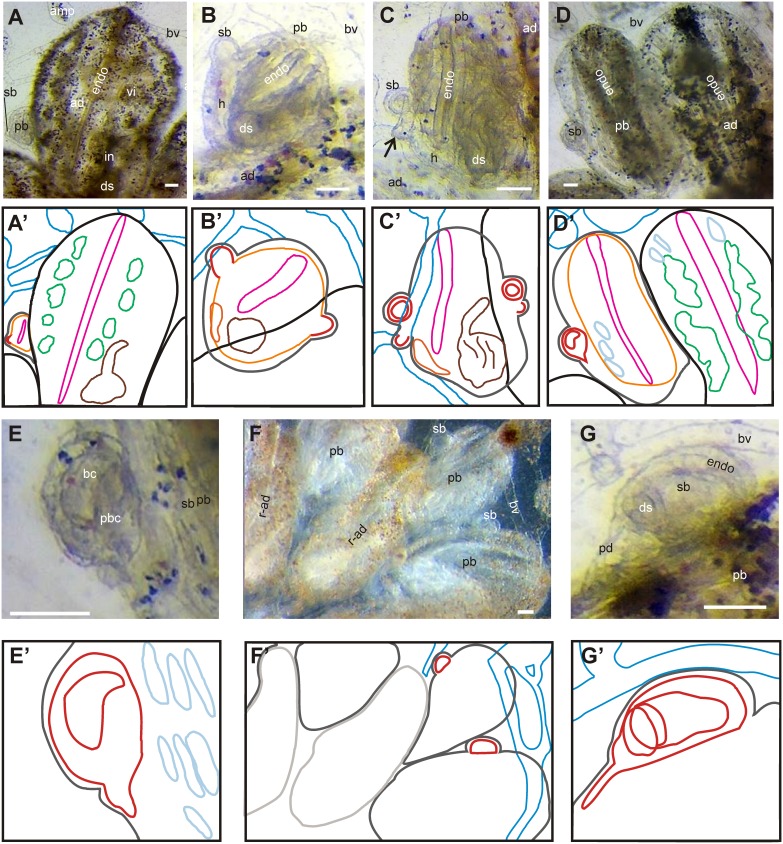
Blastogenesis in *B. schlosseri*. **A–G.** Ventral views of colonies (*i.e.*, ventral views of adults and primary buds, lateral views of secondary buds). **A’–G’.** Sketches of images A–G; black lines, adult epidermis; dark gray lines, bud and budlet epidermis; pale gray lines, regressing adult epidermis; red lines, budlet; yellow lines, branchial and peribranchial chambers in bud; orange, heart; pink, endostyle; green, ventral cell islands in adult; brown, gut; dark blue, blood ampullae and vessels in tunic; pale blue, stigmata. **A.** Adult zooid, primary and secondary buds of a colony at colonial stage 9/7/1. The primary bud (stage 7/1) has almost completed organogenesis; the secondary buds (stage 1) is recognizable as a thickening of the atrial wall of the primary bud. **B.** Primary and secondary buds of a colony at stages 9/8/2 are outlined. **C.** In stage 8/3, the heart beats in the primary bud and forms a closed sphere in the secondary bud (stage 3) that will soon begin, at stage 4 (not shown), a series of invaginations reminiscent of gastrulation. Note a small secondary bud at stage 2 (arrow) posterior to the bud at stage 3 (which determines the stage of the colony). **D, E.** Colonial stage 9/8/5 characterized by growth of the primary bud and organogenesis in the secondary bud (enlarged in E). **F, G.** In colonial stage 11/8/6, adult zooids shrink and are reabsorbed by means of apoptosis, while the primary buds mature and replace them. G: enlargement of the secondary bud. ad, adult zooid. amp, ampulla; bc, branchial chamber; bv, blood vessel; ds, digestive system; endo, endostyle; h, heart; pb, primary bud; pbc, peribranchial chamber, r-ad, resorbing adult zooid; sb, secondary bud; vi, ventral cell islands. Scale bars-100 µm.

**Figure 7:**
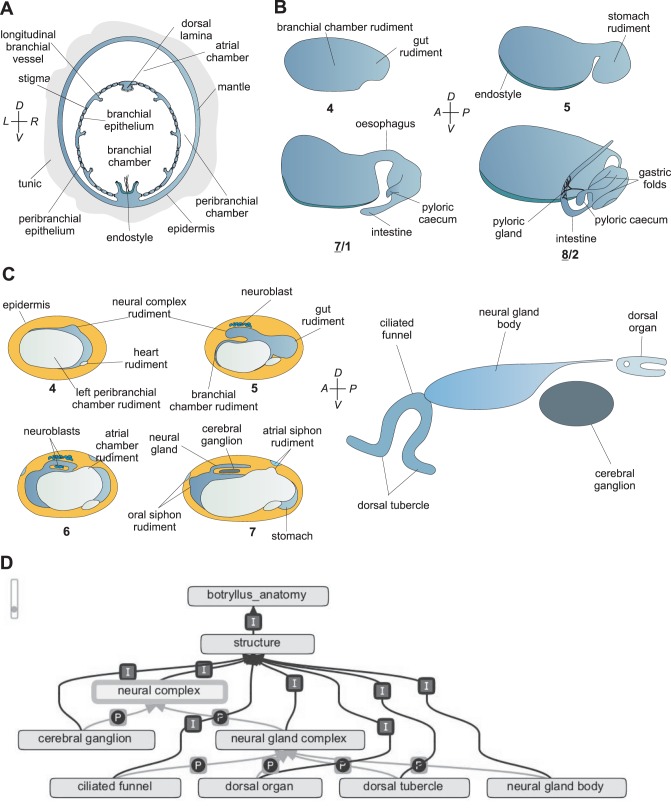
Schematised details of *B. schlosseri* blastogenesis and the relation to BODA. **A**. Schematic cross section of an adult blastozooid of *B. schlosseri* at the branchial chamber level (modified from [75]). **B**. Scheme of gut development in *B. schlosseri* bud (lateral view, anterior on the left). Each sketch shows the forming branchial chamber and the gut elongating posteriorly. Peribranchial chambers, epidermis and other organ rudiments are omitted for clarity. Stages of development are indicated below each sketch: upper sketches refer to secondary buds (stages 4 and 5), bottom sketches to primary buds (stages 7/1 and 8/2). Modified from [49]. **C**. Scheme of main morphogenetic events of neural complex development. Left: lateral view; anterior on the left; bud stages indicated by numbers. Right: scheme of the adult neural complex (modified from [47]). **D**. Graph generated by the OBO-edit editor indicating anatomical entities referring to neural complex anatomy in BODA.

The Development Ontology we propose is based on *in vivo* and histological observations following the staging method introduced by Berrill [70] and later modified by Sabbadin [6]. Most of anatomical studies describing bud development follow this staging method. According to this method, 11 stages identify the blastozooids development (see Manni et al., 2007 for a detailed description of the stages of blastogenesis; Figs. 3, 5–7).

The class developmental stage depicts the stages of blastogenesis. Each stage possesses further subdivisions expressing the relationships between the anatomical structures and their development.

Stages 1–6 characterizes the budlet development (Figs. 3, 5). The first budlet rudiment appears as a thickened disc of the peribranchial epithelium on the lateral body wall of a bud. The budlet also comprises the overlying epidermis (**stage 1**, BSD:0000012). The two epithelia delineate the **mantle** (BSA:0000097). The two epithelia (and the mantle between them) then organize into a double vesicle (an inner and an outer vesicle) **(stages 2,** BSD:0000013, and **3,** BSD:0000014) and from the inner vesicle, most of the bud structures differentiate (**stage 4**, BSD:0000015). At the same stage, the heart rudiment forms ventrally on the right side of the branchial rudiment, as a compact mass of mesenchymal cells. At **stage 5**(BSD:0000016), the stomach becomes recognizable as posterior evagination of the branchial rudiment, while a ventral extension of epidermis penetrates the tunic forming the radial vessel (**stage 6**, BSD:0000017). Stages 7–8 define the (primary) bud. The stigmata rudiments begin to form and the rudiments of the new budlets appear on the mantle of the bud at stage 7. The heart starts beating at stage 8. The adult begins its functional activity at stage 9, upon the opening of the siphons. Stage 10 refers to sexually mature zooids (see below). The sole difference with respect to stage 9 is related specifically to gonad and gamete differentiation, and as far as blastogenetic development is concerned, stages 9 and 10 are equivalent. Stage 11 refers to the take-over and is subdivided in four phases [71]: 11^1^, siphons retraction and closure; 11^2^, general shrinkage of zooids; 11^3^, further contraction of zooids and branchial dissolution; 11^4^, heart beat stops.

In the BODA, we indicated:

- secondary bud stages with a single number (from 1 to 6);
- primary bud stages with a combination of two numbers, the first one (underlined) referring to primary buds and the second to secondary buds: **7/1** (BSD:0000018), **8/2** (BSD:0000019), **8/3** (BSD:0000020), **8/4** (BSD:0000021), **8/5** (BSD:0000022), **8/6** (BSD:0000023);
- adult stages with a combination of three numbers, indicating filtering zooids (underlined), primary, and secondary buds, respectively **9/7/1** (BSD:0000024), **9/8/2** (BSD:0000025), **9/8/3** (BSD:0000026), **9/8/4** (BSD:0000027), **9/8/5** (BSD:0000028), **9/8/6** (BSD:0000029), and 11/8/6. As stated above, the takeover phase is subdivided further in four sub-stages: **11^1^/8/6** (BSD:0000030), **11^2^/8/6** (BSD:0000031), **11^3^/8/6** (BSD:0000032), **11^4^/8/6** (BSD:0000033).

Sometimes bud and budlet stages are indicated with the combination of the three numbers, underlining the second or third number, respectively.

The stage of the colony is indicated by three numbers corresponding to the stage of each generation: the first to adult filtering zooids, the intermediate to primary buds, and the last to secondary buds. 11/8/6 (or 11/9/6, when the newly developed adult coexists with the regressing adult and the budlet) indicates the take-over stage.

BODA also allows the comparison between Sabbadin’s method and the Watanabe (1953) staging method [72], subsequently modified by Lauzon et al. 2002 [38] (Fig. S1). These staging methods are introduced as “synonyms” of each Sabbadin stage. The Watanabe staging method distinguishes four recurrent phases (A to D), the latter corresponding to takeover (see [5], for a detailed comparison among staging methods).

### *Botryllus schlosseri* Organization and the Anatomy Ontology

The Botryllus_anatomy class uses three high level terms to construct the Anatomy Ontology: **cell** (BSA:0000035), **organism** (BSA:0000038) and **structure** (BSA:0000037). The “cell” branch includes the **blood cell** (BSA:0000128) and the **tunic cell** (BSA:0000043). The “organism” branch describes the colony as a whole, considered as a “super-individual”, and the zooid. Within the branches “colony” and “zooid”, all the anatomical entities are listed in alphabetical order (as in the high level term “structure”). Each entity is linked to the developmental stages of blastogenesis by means of the following “relations”: *develops from*, *end stage*, *included in*, *part of*, *preceded by* and *start stage* (see successive paragraph). Each anatomical entity has been defined according to the Annotated Glossary by Kott [73], [74] and other relevant papers on ascidian anatomy [75].

Further subdivisions define the colony into the **tunic** (BSA:0000042), the **colonial circulatory system** (BSA:0000044) and the **system** (BSA:0000049). The latter is a star shaped group of two to several filter feeding, adult **blastozooids** (BSA:0000050) with a central **common** **cloacal siphon** (BSA:0000126), into which individual **atrial siphons** (BSA:0000056) open. The colonial circulatory system consists of a network of vessels of epidermal origin, branching from the zooids and buds, crossing the tunic, and connecting to a marginal vessel, which runs along the periphery of the colony [76]. The vessels terminate toward the tunic surface in oval-shaped blind ends: the **blood ampullae** (BSA:0000045).

In BODA, the blastozooid is an entity which is *part of* a system, together with the common cloacal siphon. The latter is a structure formed by the convergence and fusion of the adult blastozooids at the centre of the system, therefore it belongs to all the adult blastozooids forming the system. The main anatomical entities of *B. schlosseri* are summarized in Fig. 3.

### Relations between Anatomy and Development in the Ontology

The class development entity comprises the terms: **meta_period** (BSD:0000000), **period** (BSD:0000006) and **stage** (BSD:0000011). The meta_period lists five high level terms: **pre-embryonic development** (BSD:0000002), **embryonic development** (BSD:0000003), **metamorphosis** (BSD:0000004), **post-metamorphosis/oozooid** (BSD:0000005) and **blastogenesis** (BSD:0000001).

The **blastozooid of first generation** (the adult) (BSD:0000009), the **blastozooid of second generation** (the bud) (BSD:0000008), the **blastozooid of third generation** (the budlet) (BSD:0000007) and **the zooid regression** (BSD:0000010) are entities comprised in the meta_period blastogenesis and also in the entity period.

The ontological relationships between each anatomical entity and its development have been built based on copious studies produced over the last 60 years. This includes descriptions of the development of specific organs such as the digestive system [49], [77], [78], the circulatory system [10], [48], [76], [79], the blood [21], [71], the heart [80], the branchial basket [81], [82], and the nervous system [7], [47], [50], as well as descriptions of biological processes such as sexual reproduction [46], [83]–[85] and take-over [42], [86], [87].

#### The blastozooid of third generation: the budlet or secondary bud

The development of the blastozooids (from the appearance of the secondary bud to its regression) covers three colony life-cycles and, at a temperature of 18°–19°C, lasts around three weeks (Fig. 5) [6], [64].

In BODA, the blastozooid of the third generation *starts* at stage 1 as a small disc-like thickening of the peribranchial epithelium (*i.e.*, “epithelium delimiting the peribranchial cavities”), overlaid by **epidermis** (BSA:0000073). The bud primordium folds into a hemisphere (stage 2), and then its inner peribranchial layer folds into a sealed vesicle enclosed by an epidermal vesicle (double vesicle stage, stage 3). From this stage, the bud remains connected to the parent by a short epidermal peduncle (BSA:0000127). Intermediate stages can also been recognized and used to better define these early developmental phases: 1+, initial arching of bud primordium; 2+, skewing of the hemisphere from its lateral orientation toward the anterior end of the parent; and 3+, elongation and expansion of the inner vesicle along the anteroposterior axis of the parent.

During budlet development (Figs 3, 5, 6), **heart** (BSA:0000088), **gonad** (BSA:0000079), **blood sinuses** (BSA:0000057), **body wall muscles** (BSA:0000058), **neural complex** (BSA:0000099) and **nerves** (BSA:0000098) *develop from* mesenchymal cells and are located in the mantle. In the inner vesicle, two long parallel invaginations of the prospective ventral side grow dorsally, to divide the original, inner vesicle into a central **branchial cavity** (BSA:0000113) flanked by two **peribranchial cavity** (BSA:0000114). A dorsal median thickening of the inner vesicle represents the region from which the neural complex, **cerebral ganglion** (BSA:0000100) and **neural gland complex** (BSA:0000101), later develop. A medio-posterior evagination of the inner vesicle represents the rudiment of the gut. The heart primordium appears in the form of a compact mass located in the mantle, ventrally on the right side of the branchial vesicle, close to the gut rudiment. Therefore, the branchial and peribranchial chambers, the neural complex, the gut and the heart *start* at stage 4 (Fig. 7 B,C). Successively (stage 5), the **endostyle** (BSA:0000064) begins to form on the branchial ventral surface (Fig. 7 B). The gut rudiment sprouts from the posterior part of the central branchial cavity and forms a rounded evagination, *i.e.* the **stomach** (BSA:0000123) rudiment (Fig. 7 B). Its base progressively narrows, and the **esophagus** (BSD:0000074) becomes recognizable as a dorsal canal between the branchial chamber and the stomach. At this stage, the heart primordium becomes a roundish vesicle, which progressively elongates into a tubular structure defined by a single layer of cubical cells. At stage 6, the peribranchial cavities progress to completion *i.e.* they come into contact with one another dorsally and posteriorly, and fuse into the **atrial cavity** (BSA:0000056) (Fig. 7 C). The **oral** (BSA:0000106) and **atrial** (BSA:0000051) **siphon** rudiments are also recognizable (Fig. 7 C). During these morphogenetic movements, the peribranchial epithelium follows gut growth, and form a series of perivisceral **blood sinuses** (BSA:0000057). Two evaginations of the stomach wall grow backward, bending to the left side of the branchial basket: the main evagination forms the **intestine** (BSA:0000129) rudiment; the smaller one forms the **pyloric caecum** (BSA:0000124) and the **pyloric gland** (BSA:0000125) rudiments. The intestinal evagination grows towards the cloacal siphon. The neural gland opens anteriorly in the prospective **prebranchial pharynx** (BSA:0000117). Posteriorly, the neural gland tube extends over the forming atrial cavity. The epithelial wall of the vesicular heart begins to invaginate along a dorsolateral line, forming the myocardial folds. The two lips of the myocardial folds divide the external **pericardium** (BSA:0000090) from the internal **myocardium** (BSD:0000089). At stage 6, the primordium of the radial vessel appears as a ventrally located evagination of the bud epidermis.

#### The blastozooid of second generation: the bud or primary bud

From stage 6 to 7, the budlet rotates counter-clockwise, assuming the same antero-posterior and dorso-ventral axis of its parent zooid. At stage 7/1, the branchial and peribranchial epithelia merge in thick zones aligned on a stereotyped pattern, forming the stigmata primordia. At the same time, the primordia of the three pairs of **longitudinal branchial vessels** (BSD:0000077) become recognizable as the thickened folds of the branchial epithelium. The **dorsal lamina** (BSA:0000062) is now recognizable on the roof (dorsal side) of the **branchial sac** (BSD:0000111). The pyloric gland rudiment is now split into two branches, each one bearing smaller branches which flank the intestinal wall and forms the rudiment of tubules and ampullae of the gland. The original connection with the stomach wall becomes the gland duct, which opens at the base of the pyloric caecum. The stomach wall begins to fold longitudinally. The cerebral ganglion is now identifiable as a distinct cell mass, ventral to the neural gland rudiment. The first nerves become recognizable in the bud, derived from branching of the two couples of anterior and posterior roots emerging directly from the ganglion.

Stage 8/2 is characterized by a beating heart. Initially, the heart beat is slow (stage 8′), but progressively reaches its normal rhythm (stage 8″) [69], [88]. It is coordinated by the heart of the adult generation, since it reverses the direction of its contractions soon after cardiac reversion has taken place in the adult. The neural gland rudiment begins to separate from the **dorsal organ** (BSA:0000103), homologous of the dorsal strand typical of other ascidians (see [47]).

During stage 8/3, branchial and peribranchial chambers become connected via a perforation of the stigmata which progresses from front to back. The rudiments of the oral **velum** (BSA:0000110) and **tentacles** (BSA:0000109) are recognizable as short evaginations of presumptive oral epithelium. The cerebral ganglion and the neural gland begin cytodifferentiation [47].

Successively (stage 8/4), in the branchial basket the stigmata enlarge and extend along the antero-posterior axes and assume an elliptical shape. At stage 8/5 the cells forming the stigmata narrow their apical side and elongate around the **gill slits** (BSA:0000075), while numerous rudimentary cilia begin to form and extend into the branchial fissure. These cilia progressively elongate and arrange themselves into a single row per cell. The gut regions become better defined: the stomach folds deepen, and the pyloric gland extends so that three intestinal regions, **proximal intestine** (BSA:0000096), **mid intestine** (BSA:0000095) (encrusted by the pyloric gland) and **distal intestine** (BSA:0000094), are now recognizable. At this stage, the peripheral nervous network reaches its maximal complexity [7].

At stage 8/6 buds are ready to substitute their adult parental zooid, which undergo take-over. This stage triggers the change of generation, *i.e.* stage 11/8/6, characterized by siphon aperture in buds (which become the new filtering adults at stage 9) and the full absorption of the “old” parental zooids.

#### The blastozooid of first generation: the filter-feeding adult stage

Two main morphogenetic processes occur in adults; the beginning of their filtering activity (stage 9/7/1) and their re-absorption (stage 11/8/6). After several blastogenic cycles following larval metamorphosis, the **ventral cell islands** (BSA:0000128), which are a cluster of cells flanking the endostyle appear in the mantle of the adult zooids, (stage 9/7/1 or 9/8/2). In stage 9/8/5, the ventral cell islands are no longer visible in adult zooids. Their cells migrate into the colonial circulatory system prior to takeover to colonize new sites belonging to the buds of the subsequent generation [30], [41].

The oral siphon opens firstly (9^1^), followed shortly after by the atrial siphon (9^2^); in succession, the adults of a system modify their position and meet one another to form the common cloacal siphon (9^3^). Water flows into the branchial chamber propelled by branchial ciliary activity, and both active oxygen exchange and feeding begins [69]. The dorso-posterior regions of the blastozooids grow backward and rise to form the **cloacal lips** (BSA:0000055) defining the common cloacal chamber. Stage 10 defines a colony with sexually mature zooids. Since sexual maturity occurs in the colony after several blastogenic cycles in healthy colonies [67], this stage is considered separately (see below).

#### The zooid regression: the take-over

The take-over is initially identified by the unresponsiveness of the oral siphons to mechanical stimuli followed by the closure of the oral and then the cloacal siphons, and the consequent end of filtration (stage 11^1^). The zooid then undergoes a general shrinkage and agglutination of branchial cilia (stage 11^2^), and while resorption continues, a functional heart persists (11^3^). The heart stops beating and the zooid becomes a piknotic vesicle in the center of each system (stage 11^4^). Stage 11/8/6 represents the *end stage* for the anatomical structures within the adult blastozooids.

### Relationships between Sexual and Asexual Reproduction: the Gonads Development

In *B. schlosseri*, new colonies go through an asexual juvenile phase and reach full sexuality after several blastogenetic generations, starting with the development of **testis** (BSA:0000085) [64]. Bilateral gonadal primordia *starts* in the secondary bud as clumps of undifferentiated cells on either side of the inner vesicle at stage 3 [83]. The medial portion of this primordium differentiates into a testis, in the form of a coherent structure which reaches maturity in adults and discharges most of the sperm one to two days after ovulation, thus avoiding self-fertilization [83], [89]–[91]. The lateral portion of the gonadal primordium becomes an **ovary** (BSA:0000080). Only one or a few **eggs** (BSD:0000081) ripen on either side. Oocytes appear in secondary buds, ripen in the primary buds and ovulate when the primary buds are about to pass to the adult stage (10/7/1). Fertilization occurs just after the siphon opens, and the larvae are released when the adults are almost at the end of their life-cycle.

During its growth, the oocyte is surrounded by three **egg envelopes** (BSA:0000082): test cells, inner and outer follicle cells, all deriving from primary follicle cells [84], [85]. Test cells are separated from follicle cells by a fibrous layer, the vitelline coat (or chorion). At ovulation, the egg hatches from the outer follicle cells, which may persist for several hours as an analogue of the vertebrate *corpus luteum*. The inner follicle cells participate in forming the **placental cup** (BSA:0000084), together with the parental peribranchial and oviductal epithelia, and the embryo is held in the peribranchial chamber [46].

Although in BODA, the gonad is an anatomical structure *part of* the blastozooid, it is important to note that the germinal cells should be considered *part of* the colony. It has long been known that oocytes and male elements can be captured by the bloodstream within the tunic vessels and conveyed to other zooids. Their recycling through successive generations has been regarded as the necessary condition for their maturation [25], [83].

### Exploiting the BODA: the Development of the Nervous System

The study of the development of the nervous system offers a good example on how to exploit the potential of BODA (Fig. 7 C–D). In the oozooid, the nervous system is derived directly from the anterior neural plate [92], while in the blastozooid it originates from the inner vesicle of the bud [47]. Nevertheless, it has the same organization in both oozooid and blastozooid. As some adult neural components show a placodal derivation in embryogenesis [8], [93], [94] *B. schlosseri* offers an interesting opportunity to understand how structures which are fundamental for chordate evolution are produced by an alternative developmental process (blastogenesis).

The neural complex is defined as “constituted of the cerebral ganglion and the neural gland complex (dorsal to the former); it is located in the dorsal mantle between the two siphons”. The neural complex is *part of* the blastozooid and comprises two subdivisions; both are *part of* the neural complex. The subdivisions are the cerebral ganglion (“organized in a cortex of neuronal somata and an inner medulla of neurites continuous with nerves”) and the neural gland complex. The neural gland complex is a “gland in the form of a blind sac, located in the dorsal mantle beneath the epidermis and opened in the branchial chamber; it is located in the dorsal mantle between the two siphons”. The neural gland complex entity possesses, as subdivisions, the **ciliated funnel** (BSA:0000102) (the “funnel-like duct of the neural gland opening into the prebranchial pharynx”), **neural gland body** (BSA:0000104) (the “elongated gland body constituted of spongeous cells”) and the dorsal organ (the “organ posterior to the dorsal gland body, homologous to the dorsal strand”).

The neural complex is *part of* the blastozooid and it *develops from* the “**dorsal tube**” (BSA:0000063). The latter is the “neural complex rudiment”. The dorsal tube is *part of* the blastozooid (as the neural complex). It *develops from* the bud inner vesicle; its *start stage* is 9/8/4, when the inner vesicle begins morphogenetic movements. The dorsal tube’s *end stage* is 7/1; this is the *start stage* of the two entities deriving from the dorsal tube (*i.e.*, the cerebral ganglion and the neural gland complex). The three subdivisions of the neural gland complex, the ciliated funnel, the neural gland body and the dorsal organ, have as a *start stage* the stages 9/8/3, 9/8/3 and 9/8/2, respectively. The *end stage* of the neural complex (and all its subdivisions) is stage 11 (zooid regression).

Burighel and collaborators (1998) [47] have described in detail the development of the neural complex during the blastogenesis of *B. schlosseri*. The sources and the bibliographical details regarding the morphogenesis of the central nervous system, and all the anatomical entities listed in the Ontology, can be found in the literature cited in the BODA.

## Discussion

Using the rules defined by the Open Biomedical Ontologies (OBO) Foundry [65] we have built the BODA with the final goal of gathering and formalizing the existent data about the anatomy and blastogenesis of *B. schlosseri*.

The BODA features will allow the users: 1) to easily search and identify anatomical structures in colony and zooid, 2) to define the correct developmental stage and 3) to follow the morphogenetic events of a tissue and/or organ of interest throughout asexual development.

### Why an Ontology for *Botryllus schlosseri* Asexual Reproduction?

*B. schlosseri* reproduces both sexually and asexually. During sexual reproduction, the fertilization and the development are internal [46] and only a small amount of eggs/embryos per zooid reach maturation. Embryogenesis lasts five days at 18–19°C and late developmental stages are mainly identified by means of tail length of embryos that grows below the vitelline coat. There is an established correlation in timing between sexual and asexual development. When the colony is at stages 10/8/2, 10/8/3, and 10/8/4–5, the embryo respectively reaches: i) the early tail bud stage about 3 days before hatching with the tail making a three-quarter turn around the trunk, ii) mid-tail bud stage about 2 days before hatching with the tail making one turn around the trunk, and iii) late tail bud stage about 1 day before hatching with the tail at its maximum extension (1.5 turns around the trunk) [92]. It appears clear that these staging parameters are not sufficiently defined, neither temporarily nor morphologically, for a developmental ontology.

Embryos are brooded inside the parental peribranchial chamber, attached to it by a placental-like cup, and after *in vitro* fertilization, embryos usually do not survive the first mitoses, suggesting that the contribution of the parent is essential for embryonic development. As a consequence, *B. schlosseri* embryogenesis is a more difficult and complex study compared to solitary, oviparous ascidians with external fertilization, such as *C. intestinalis*. For these reasons, the embryonic development, well studied in *C. intestinalis* where complete cell lineage has been determined and precisely temporized [61], has not yet been properly described in *B. schlosseri*.

Conversely, easy access to an ontology illustrating asexual reproduction and valorised by a well-characterized description of the anatomy and development of budding will be an important resource for the study of the nature of the asexual reproduction in *B. schlosseri. B. schlosseri* has the recognized potential to facilitate basic research on stem cells [11], [13], [24], [25], [30], [32], and can provide insights on the evolution, and loss, of regenerative abilities in vertebrates. At the moment, migrating cells belonging to ventral cell islands represent a good candidate to explore these hypothesis [11], [13], [25], [30]. Recent studies suggest that ventral cell islands contain different kinds of cells. Their function seems to be linked to a phagocyte and/or to a putative stem cell dynamic [30], [41]. The development of transgenic animals will be crucial to determine the origin and differentiation of tissues in the new bud, and to clarify whether they originate from stem cell driven processes and/or trans/de-differentiation of adult somatic epithelia. The addition of these studies to BODA will provide a valuable tool for scientists interested in the origin of cells, tissues and the biology of regeneration.

It is also notable that sexual and asexual ontogenies in *B. schlosseri* give rise to essentially the same adult body plan [37], [95]. For this reason, *B. schlosseri* has been used to investigate similarities and differences between two development pathways, within the same species [8], [44], [93], [95], [96]. Recent studies have shown that genes involved in the differentiation of neural placodes in vertebrates are expressed in both the developmental pathways of *B. schlosseri* [93]. This suggests that fundamental genes for the differentiation of the vertebrate sensory system were present in the common ancestor to tunicates and vertebrates, and were recruited in the evolution of asexual development of tunicates. Therefore, the ontology of a chordate species able to reproduce both sexually and asexually turns out to be particularly relevant in a comparative and evolutionary perspective.

In existing ontologies, the OBO Foundry’s suite and principles have been adopted to describe the ontology and development of asexually reproducing Eukariota, including plants [97], social amoeba [98] and yeast [99]. Ontologies for animal species commonly refer to embryogenesis and related anatomy. Exceptions occur in the Kingdom Animalia - for example, in one species of porifera, asexual fragmentation is simply introduced as an entity (*i.e.*, term) related to biological proprieties (https://code.google.com/p/porifera-ontology/). In the polychete *Platynereis,* the ontology is not yet available for its asexual morph (the epitoke), but the developmental stages have been described, (http://4dx.embl.de/platy/). Therefore, BODA represents the first fully built anatomical and developmental ontology resource available for an asexual reproducing bilaterian, and is an encouraging starting point for the formalization of other ontologies based on “non-canonical” modes of development, such as blastogenesis.

### Structure of BODA, Its Implementation and Integration

BODA can be accessed freely from the Ontology Bioportal (http://bioportal.bioontology.org/ontologies/BODA) [66] and/or NISEED platform (Fig. 4), or downloaded from OBO Foundry portal [65], from the Ontology Bioportal [66] or from the Tunicate Portal (http://www.tunicate-portal.org/wordpress/?page_id=145).BODA is integrated on the NISEED platform [63] *i.e*. the reference portal in the tunicate community, which has already assembled data from several ascidian species such as *Ciona intestinalis*, *Halocynthia roretzi* and *Phallusia mammillata*. The integration on this common platform facilitates comparative analysis across species. In this respect, the BODA is the first ascidian ontology to be enriched with definitions of anatomical entities. Definitions were acquired consulting a number of publications (cited in the Results section). Some definitions specifically describe structures properly belonging to *B. schlosseri*, but many are general and can be used as a guideline by researchers working on other ascidians.

For example, BODA does not contain some anatomical entities included in *C. intestinalis* Ontology, such as the dorsal strand and the dorsal strand plexus, simply because they are not present in *B. schosseri*. *Vice versa*, BODA lists anatomical entities that are not possessed by *C. intestinalis* (such as the dorsal organ or the colonial circulatory system). Other entities, even if possessed by *C. intestinalis,* are not inserted at the moment in the *C. intestinali*s Ontology (e.g., sperm, egg, coronal organ); this is mainly due to the fact that *C. intestinalis* Ontology is based on embryonic cell lineage. As a consequence, the anatomical entities for which the exact embryonic derivation is not known were not included in the Ontology.

The structure of BODA allows the system to be easily implemented with new heterogeneous data and metadata. For example, sexual development definition, gene expression profiles, transcriptomic data, phenotypes generated by knock-down experiments, drug treatment tests and all the related literature could be easily introduced once available. This will facilitate the ability to find integrated information. For example, by editing keywords or searching for sequences, a specific gene can be easily related to the anatomical entities and developmental stages in which it is expressed, representing its spatio-temporal expression profile.

In conclusion, we encourage the tunicate community to use BODA, to provide feedback, and to update this resource with an upload of its latest discoveries.

## Supporting Information

Figure S1 ***Botryllus schlosseri*** **staging flowchart for asexual development.** Flowchart for asexual development of *B. schlosseri* that help to individuate *in vivo*, under a dissection microscope, the stage of the colony, and permit also to easily compare the staging methods by Sabbadin [6] and Lauzon [38].
(PDF)
