# Supplementary figures and images for: Ontology for the Asexual Development and Anatomy of the Colonial Chordate Botryllus schlosseri
Source: PLoS One. 2014 May 1;9(5):e96434. doi: 10.1371/journal.pone.0096434 (PMC4006837; doi:10.1371/journal.pone.0096434)

# *Botryllus schlosseri* staging flowchart for asexual development

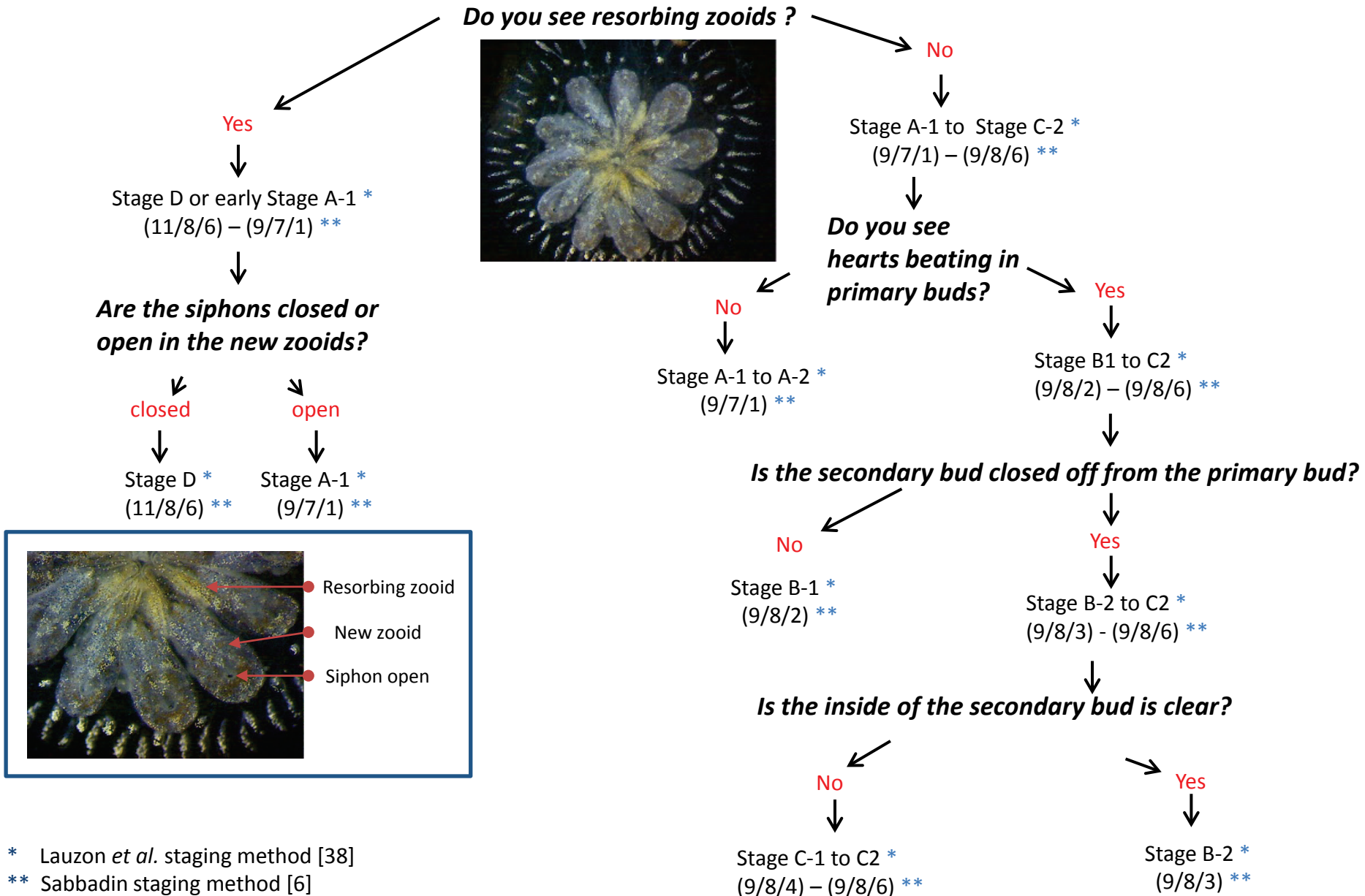

Supplement: Figure S1 — Botryllus schlosseri staging flowchart for asexual development. Flowchart for asexual development of B. schlosseri that help to individuate in vivo, under a dissection microscope, the stage of the colony, and permit also to easily compare the staging methods by Sabbadin [6] and Lauzon [38]. (PDF) [file pone.0096434.s001.pdf]
